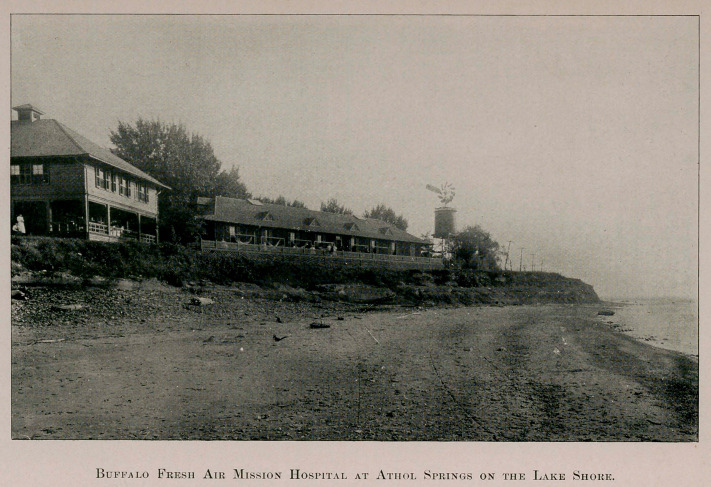# Concerning the Symptomatology and Treatment of Infantile Diarrhea at the Buffalo Fresh Air Mission Hospital1Read at the twenty-eighth annual meeting of the Medical Association of Central New York, at Syracuse, October 15, 1895.

**Published:** 1896-01

**Authors:** Irving M. Snow

**Affiliations:** Buffalo. N. Y., Instructor in diseases of children, University of Buffalo; physician to Buffalo Fresh Air Mission Hospital; member American Pediatric Society.


### BUFFALO MEDICAL JOURNAL.

VOL. XXXV. JANUARY, 1896. No. 6.

### Original Communications.

### CONCERNING THE SYMPTOMATOLOGY AND TREATMENT OF INFANTILE DIARRHEA AT THE BUFFALO FRESH AIR MISSION HOSPITAL.[1]

By IRVING M. SNOW, M. D., Buffalo, N. Y.,

Instructor in diseases of children, University of Buffalo; physician to Buffalo Fresh Air Mission Hospital; member American Pediatric Society.

THIS paper will deal with the experience of the writer in the treatment of infantile diarrheas at a lake shore hospital. The object is to describe some of the many phases of the dyspeptic and inflammatory diarrheas of children and to discuss their mechanical, dietetic and climatic treatment. My patients were treated at the Buffalo Fresh Air Mission Hospital, which is charmingly situated on the lake shore at Athol Springs, ten miles from Buffalo. The buildings are new, conveniently planned and stand on the edge of a bluff overhanging Lake Erie. The institution is fully equipped with apparatus, possesses resident physicians and trained nurses, and is reached in twenty-five minutes from the city.

By the courtesy of my colleague, Dr. Dewitt H. Sherman, I am able to report the entire service of the hospital. We are accustomed to divide our cases into two classes: 1. (*a*) Those children who are suffering from diarrhea caused by the irritation or non-assimilation of food—dyspeptic diarrhea. (*b*) Where fever, vomiting and purging were present from bacterial poisoning, but no marked inflammatory or necrotic process had taken place—mycotic diarrhea. Dyspeptic and mycotic diarrhea, forty-one cases, three deaths. 2. Inflammatory diarrhea, ileo-colitis, acute or prolonged in its course. With these cases there was pronounced intestinal lesions—congestive, inflammatory, ulcerative—ninety-three cases, fifteen deaths. Total number of cases received into the hospital in 1894-1895, 138; deaths, twenty-one; excluding the patients

1. Read at the twenty-eighth annual meeting of the Medical Association of Central New York, at Syracuse, October 15, 1895.

who died in twenty-four hours (eight), the mortality was 10 per cent.

The history of our cases was a monotonous recital of the effects of hot city air and of artificial feeding. As the patients were generally improperly fed through the entire year and as the cholera infantum season opens about the last of June or the first of July, we are inclined to believe that summer heat is the chief etiological factor. To attain its full morbific effect the high temperature must be continuous for several days and nights. Practically, here all of the cases received into the hospital in 1895 sickened in the last week in June, when the weather was unusually warm. Some of the children were at once acutely ill, the rest showed signs of failing health—gastro-enteric dyspepsia, with recurring diarrheas of more or less severity until they were removed to the hospital. The usual explanation of the derangements for the gastro-enteric tract in summer is to us sufficient, viz.: that the heat lowers the digestive powers of the child and produces poisonous changes in the food. The first heat of July is most depressing and malignant. The diarrheas of the latter summer have always seemed to the author less severe and more easily cured.

Total deaths in hospital in July, 15; August, 5; September, 1. In Buffalo, deaths from infantile diarrheas for two years, July, 429; August, 324; temperature, July, 1894, 71°; August, 1894, 67°; July, 1895, 68°; August, 1895, 69°.

It is, of course, possible that the weaker children die at the beginning of the hot season.

In reviewing the previous dietary of infants ill with diarrheal disease, we may conclude that no artificial food gives immunity from illness; even changes in hot weather from a poor food to one better may produce a diarrhea. Children sicken on condensed milk, cow's milk—sterilised and unsterilised—Liebig's and dried milk foods and broths. Moreover, the digestion seems to undergo a complete transformation in hot weather. Babies may be given articles of adult diet in cool weather and thrive. In July and August they die from the same menu. Our cases had usually received a variety of foods, tried by the mother in despair at her child's persistent vomiting, diarrhea and emaciation. The articles given were sometimes theoretically correct. Thus sterilised milk and cream or whey occasionally caused protracted dyspeptic diarrheas. There is a lowered ability to digest cow's casein in summer. Not more than 1 per cent. should be present in any food given to a child less than nine months old. After loose passages and vomiting have commenced, it is nearly impossible to make the baby digest cow's casein again until cool weather. Nevertheless, the author has seen more city children in summer thrive on cream and milk or cream and whey mixed, perhaps, with Liebig's food, than any other mixture. The various patent powdered foods, rich in starch and poor in fats, seemed sometimes to be the cause of severe exhausting diarrheas. A bottle-fed baby generally shows a loss of appetite and has a stationary or diminishing weight some days before the diarrhea begins. Errors in feeding are not always due to maternal ignorance. Women in all ranks in life are glad to receive and carry out intelligent professional advice. The physician's instructions are too often confused and show indifference and lack of knowledge. Institution children from asylums and almshouses, although apparently well fed, succumb easily to summer diarrheas. They lack a mother's constant attention and the out-of-door life enjoyed by the children of the well-to-do.

To make clean-cut classifications of the various kinds of infantile diarrheas is difficult, as acute mycotic diarrhea may change into an ileo-colitis, and the slow convalescence from an inflammatory diarrhea is the clinical picture of a dyspeptic diarrhea.

Clinically considered our cases showed the following symptoms :

(1) *Dyspeptic Diarrhea.*—A large number of infants were received with normal temperature or trifling fever. They had usually been fed on several kinds of food—cow's milk, dextrine foods, condensed milk, and the like. Their condition was fairly good. They vomited occasionally and had from five to ten stools daily for a week or two. The passages were watery, green or brown, consisting principally of the débris of food. These diarrheas were wholly due to indigestion and commonly disappeared after intestinal irrigation and careful feeding. None of these cases died ; yet it is probable that had they been left in the city many would have perished, as the history of our fatal cases showed that most of them had previously had a dyspeptic diarrhea. Hence, a mild dyspeptic diarrhea may easily become an ileo-colitis. These patients should be kept in the country and be carefully fed until cold weather.

(2) *Mycotic Diarrhea.*—This phase of gastro-enteric disease may be grafted on a dyspeptic diarrhea, or may begin abruptly in a plump, apparently healthy baby. The patient is at first fretful, the temperature rises rapidly to 104° or 105°, and there may be fifteen to twenty green or brown fetid stools in twenty-four hours. Very quickly the child becomes exhausted and the patient is brought to the hospital in a collapsed condition, with a high internal temperature. Three deaths were recorded dying with these symptoms, one with the white serous stools of cholera infantum. The convalescence from acute mycotic diarrhea is slow, the child having sometimes loose passages and irregular fever for weeks.

3. *Ileo-colitis with Marked Inflammatory Changes and Ulceration in the Ileum and Colon.*—(*a*) Ileo-colitis may commence with a fierce acute onset, continuous high temperature, frequent stools of mucus, sometimes mixed with blood ; there is frequently tenesmus, rarely persistent vomiting. The little patients sink rapidly, victims of an intense toxemia. The high fever can only be lowered by brute force, through cold baths and cool irrigations. Enormous doses of stimulants are necessary to sustain the failing heart. Our cases of ileo-colitis lasted from four to ten days. About one-third died. Those recovering made a slow convalescence, the fever and mucous stools recurring again and again. Generally from 15 to 30 per cent. of the weight was lost in four or five days. The author saw three cases of ileo-colitis in well-to-do families die with these symptoms last summer. The patients were under the best hygiene, correctly fed, and the dangerous nature of the illness was recognised early. They seemed to have received a lethal dose of some unknown poisonous germ.

(*b*) Again, intestinal inflammation may develop more insidiously. Children entered the hospital who had been ill ten to twelve days, with six to eight passages of mucus, blood and undigested food a day. They were indifferent to their food, had low irregular fever ; now and then the temperature would rise to 104° or 105°, and the number of stools would increase and diminish independent of medication or diet. The fatal cases, toward the end, had a high temperature and failing heart. They died, apparently, from a toxemia, and not from the diarrhea, which was generally moderate and not enough to cause much depression. The vagaries of the temperature chart sometimes suggested malaria, but in the most pronounced of these cases the plasmodi malariæ were not found. Most of these cases got well, yet a prognosis was a hazardous matter, as one of the most unpromising cases recovered, and a child whose improvement we regarded very complacently as a result of good treatment, died suddenly with a high temperature, although it had but three stools in twenty-four hours. Ninety-three cases of ileo-colitis, fifteen deaths.

4. We received a number of children from asylums whose records showed a high summer mortality. These babies were under weight and rachitic. There was practically no diarrhea—two to four passages of undigested food a day—they were indifferent to their food and some subject to mysterious attacks of fever.

5. Occasionally children in the last stage of atrophy were sent in. They were rachitic, generally had a bronchitis, were apathetic, difficult to feed. Two deaths were recorded from this condition. The babies had a few loose passages and died very suddenly.

6. Infantile diarrhea may be septicemic, the blood poisoning localised in the gastro-enteric tract. The author saw, in June, a case of intestinal septicemia whose chief symptom was numerous stools of mucus and blood. No case of this kind was received into the hospital.

With many of our cases the gastro-intestinal diseases were complicated by other conditions. Rachitis played an important rôle in the deterioration of the child's health. Of 138 cases in two years, 26 per cent. were rachitic. An acute illness in a rachitic baby is always a serious matter. The ability of an infant to withstand a diarrhea or a pneumonia depends often on the presence or absence of rickets. It should be remembered that there are fat rickets and thin rickets.

A convulsion during a diarrhea is a complication of evil omen. Five cases suffered from eclampsia, two died of ileo-colitis, one of mycotic diarrhea.

*Measles.*—Three patients were received who were recovering from measles; one died. French authors have repeatedly emphasised that ileo-colitis occurs as a complication, but more frequently as a sequel of measles.

*Bronchitis, Broncho-pneumonia.*—About one-fourth of our cases showed pulmonary symptoms, generally a subacute bronchitis. Four cases of well-marked broncho-pneumonia were discovered; two died. M. Sevestre has described a class of pneumonias of intestinal origin, due to infection of the pulmonary tract by intestinal bacteria. The avenues of infection are the blood or lymph channels, or, possibly, by inhalation of air polluted by the exhala tions of the stools. It is certainly a common and unfortunate event for a child with an ileo-colitis to develop broncho-pneumonia.

*Skin diseases.*—Eczema, impetigo contagiosa, scabies, were present in perhaps 15 per cent. of our cases. Involving small areas the eruption is of little consequence. If there be an universal eczema, or if there be much pustular inflammation, a diarrhea is of serious import, being probably due to septic absorption from the skin. The author saw a child, with extensive eczema since birth, die after an illness of twelve hours. During this time he had trifling fever, vomited once and had four moderately sized colorless stools. Death caused by a toxemia from absorption from the skin.

Hutinel has made some interesting observations on cutaneous infections with children. The skin is a reservoir for germs and may affect the system by absorption or inhalation. He traced several fatal diarrheas to the presence in the blood of staphylococci from impetigo or pustular eczema.

*Prognosis.*—In the light of my hospital experience the course of no infantile diarrhea can be accurately foreseen. No case is too insignificant to be neglected, no case too desperate to be abandoned. A child is fortunate who enters the summer in good condition. Babies who are under weight, who suffer from chronic vomiting or from undigested stools, will almost certainly be ill in July or August. All cases of diarrhea with persistent fever are dangerous; frequent mucous stools, whether of pure mucus or mucus mixed with blood, indicate an intense infection.

Dyspeptic diarrheas are generally amenable to treatment; mycotic diarrheas do well if not overfed. The ileo-colitis, of slow onset and irregular fever, usually offers a favorable prognosis. It is, of course, understood that the prognosis is always better among the children of the well-to-do than among the wasted, neglected offspring of the city poor. Beyond the second year there is a relative immunity against severe diarrheal disease. Only four of our twenty-one fatal cases were over two years old.

*Treatment.*—Nearly all of the cases received at the Buffalo Fresh Air Mission Hospital had been under treatment several days. Frequently the children were sent out moribund; thus eight of twenty-one fatal cases died in twenty-four hours. A large number of babies arrived collapsed, with excessively weak heart and high temperature. They were bathed, freely stimulated, put in a quiet place and generally after a time revived.

The diarrhea treatment is (1) dietetic, (2) medicinal, (3) mechanical or local.

(1) Practically the only medicines producing positive effects were opium and bismuth subnitrate. The deodorised tincture of opium was used in small doses, frequently repeated. Opiates should be cautiously given to children with a high temperature or to wasted apathetic infants. Bismuth was prescribed in massive doses, given at short intervals. The vegetable and mineral astringents were not used by the mouth. After repeated trials of the so-called intestinal antiseptics—salol, naphthaline, beta-naphthol, beta-naphthol-bismuth,—the staff have grown sceptical as to their utility. Calomel and sodium salicylate may be excepted from this list.

Great reliance was placed upon intestinal irrigation. In a large number of cases of dyspeptic and inflammatory diarrhea this procedure seemed positively curative. The entire large intestine is evacuated and cleansed. The hot currents of water in the intestines have a soothing, sedative effect upon a restless, fretful baby. There is a direct antiseptic and astringent action upon the congested and ulcerated mucosa of the colon. Borax was mixed in the water if the feces contained much mucus. If astringents were indicated, 1 to 2 per cent. solutions of tannic acid were employed. Often saturated solutions of boric acid were given if the diarrhea was toxemic. The amount of fluid used in the irrigation was large, two to four quarts once or twice a day. Permanent depression was not observed after this treatment ; in fact, the irrigation seemed the best mode of overcoming collapse. Generally, after the intestinal flushing, the number of passages would diminish and the diarrhea, *per se*, was not a difficult symptom to treat.

*Vomiting.*—Vomiting was most frequent in acute ileo-colitis and in mycotic diarrhea and in chronic gastric dyspepsia. All infants with diarrhea vomit occasionally. We did not consider it a difficult symptom to treat, except in the habitual regurgitation of infantile atrophy. The stomach was washed out. After some hours a peptonised cream and milk or cream and whey, well diluted, or chicken broth was administered in small quantities. Calomel is an agent of some value to an irritable stomach.

*Fever.*—Of all problems in the diarrheal diseases of children, the successful treatment of high temperature is the most difficult to solve. A persistent high temperature, 104° to 105°, in an ileo-colitis, is a sign of impending danger. A steadily rising temperature in chronic diarrhea or in dyspeptic, wasted infants, is of grave significance. Excepting the administration of sodium salicylate, useful as an intestinal antiseptic and antipyretic, the treatment of fever in the hospital was by hydrotherapeutics. The temperature could be reduced in three ways :

1. By sponging with cold water. The sponge baths could be frequently repeated if the temperature arose again. This procedure was soothing and generally relieved the fevers of dyspeptic diarrhea or chronic ileo-colitis. Failing with this, we had recourse to cold baths or cool irrigations.

2. The cold baths were useful in acute ileo-colitis or in mycotic diarrheas. Our patients were placed in water at 95° and the bath was then lowered to 85° or 80°. The temperature was frequently taken in the rectum and the patient removed from the bath when the thermometer recorded 101.5°, as the fever sunk rapidly after removal from the water. If the patient seemed depressed it was stimulated by the mouth or hypodermically. No serious after-effects were observed, although the radial pulse was apt to become thready from vasomotor contraction. The baths lasted from five to seven minutes.

3. Cold irrigations. Children with high rectal temperature, collapsed, with cold extremities, were stimulated hypodermically. The trunk and extremities were wrapped in hot flannels, and a cool irrigation, 80° to 95°, was given. The water was hot when it returned from the bowels. The temperature rapidly sunk and often did not again ascend.

*The Question of Stimulation.*—To nearly all cases of acute or chronic diarrhea, where alcohol did not cause vomiting, whisky was freely administered. The effects were usually beneficial; indeed, most of our cases were received in a condition of extreme exhaustion. Strophanthus, strychnia and caffein were frequently employed. Strong coffee often had a happy effect when the apathetic condition of the child seemed to contraindicate alcohol. Hypodermic stimulation was of great service in sudden cardiac depression or when we apprehended vomiting. Hypodermic medication is too little used in children. An apparently moribund infant may be saved occasionally by a timely hypodermic of strychnia, digitalis, caffein or whisky.

*Feeding.*—The proper feeding of infants, with diarrheal disease, is the key of the whole treatment. Intestinal lesions progress with the administration of indigestible food. They often heal with great rapidity if correct dietetics be employed. These children, nevertheless, often show great repugnance to food. The food prescriptions which seemed of greatest value at the Buffalo Fresh Air Mission hospital were peptonised milk, broths and beef juice. Mixtures of cream and milk, peptonised and well diluted, and cream and whey were usually well taken and easily assimilated. They are best cautiously prescribed at first, one or two feedings a day, until a tolerance is established. Fresh beef juice may be administered at the same time or separately. Prepared according to Cheadle's formula, it contains 8 per cent. of albuminoids. It was given to the children pure or diluted with three to four times its bulk of water.

*Value of Country Air.*—It is nearly impossible to keep an artificially fed infant well during the hot season, except in the country. It is equally difficult to cure dyspeptic or inflammatory diarrhea during July or August in a city house. In the country the appetite is apt to increase and all of the symptoms usually ameliorate, yet the improvement is not always instantaneous, magical. Ileo-colitis and cholera-infantum are not immediately cured by removal from the city.

We think that physicians often err in stating to parents that their baby will immediately recover if sent to the lake shore or a farm, and that nothing else is necessary. The result is often disastrous and disappointing, for, with even Eden-like surroundings, the little patient may be harassed by recurring fever, vomiting and diarrhea. Prolonged residence is necessary. The fresh air should be associated with skilful nursing and medical attendance. Hence the great value of lake shore hospitals like the institutions at Athol Springs and Charlotte.

476 Franklin Street.

## Figures and Tables

**Figure f1:**